# Both sides of the story: comparing student-level data on reading performance from administrative registers to application generated data from a reading app

**DOI:** 10.1140/epjds/s13688-021-00300-y

**Published:** 2021-08-19

**Authors:** Bent Sortkær, Emil Smith, David Reimer, Stefan Oehmcke, Ida Gran Andersen

**Affiliations:** 1 Danish School of Education, Aarhus University, Aarhus, Denmark; 2 Department of Computer Science, University of Copenhagen, Copenhagen, Denmark

**Keywords:** Administrative data, Application generated data, Reading speed, Reading speed progress, Machine learning, Reading test, Learning analytics

## Abstract

The use of various learning apps in school settings is growing and thus producing an increasing amount of usage generated data. However, this usage generated data has only to a very little extend been used for monitoring and promoting learning progress. We test if application usage generated data from a reading app holds potential for measuring reading ability, reading speed progress and for pointing out features in a school setting that promotes learning. We analyze new data from three different sources: (1) Usage generated data from a widely used reading app, (2) Data from a national reading ability test, and (3) Register data on student background and family characteristics. First, we find that reading app generated data to some degree tells the same story about reading ability as does the formal national reading ability test. Second, we find that the reading app data has the potential to monitor reading speed progress. Finally, we tested several models including machine learning models. Two of these were able to identify variables associated with reading speed progress with some degree of success and to point at certain conditions that promotes reading speed progress. We discuss the results and avenues for further research are presented.

## Introduction

In the past decades, governments around the world have increasingly engaged in the assessment of student performance. The OECD’s Programme for International Student Assessment (PISA) which tests students in domains such as their mathematical skills and reading ability has since its inception in the year 2000 become a global phenomenon and is a prime example of this trend (see [1]). The growth of accountability and student testing systems is thus a major global trend and can also be witnessed at the national level (for example [2]).

Another major trend in education is digitization and availability of big data [3]. Students and teachers are increasingly using learning platforms, learning apps or other digital tools resulting in the generation of an enormous amount of data.

Also in Denmark, these two aforementioned trends coincide. Denmark has implemented an extensive national level testing system where students are repeatedly tested in various domains [4]. At the same time, the level of digitization is very advanced in an international comparative perspective and Danish students are among the top performers in terms of computer and information literacy in Europe [5]. Apps that allow easy access to online reading, mathematical problem solving or the administration of homework are used in most schools across the country very frequently [6]. The pros and cons of both trends, the increased intensity and use of tests and digital platforms have been the topic of some debate. Critiques of the national tests in Denmark have pointed to the fact that the administration and maintenance of the testing system is too costly, test-taking is detrimental for student well-being and takes time away from teachers and students that could be used on other activities [7, 8]. Proponents of these tests have pointed to the fact that tests are more objective than teacher assigned grades and allow for better comparability of student performance across classes, schools and over time [9].

The advantage of a frequent use of digital tools has long been a topic for research. One meta-analysis report an overall positive effect for the use of mobile devises in classroom teaching in terms of learning outcomes [10]. However, the use of digital tools in the context of teaching and learning activities has also been met with skepticism. The students’ engagement in a digital text is in fundamental ways different from reading a print book and the difference in learning outcomes is yet unresolved [11]. Furthermore, given that students can be tempted to use their digital device for numerous activities at the same time, many of them potentially not related to learning, researchers often associate the increased use of mobile devices with student problems in terms of maintaining concentration [12].

A potential advantage of the increased use of digital tools is, however, that data generated by the use of the apps gives teachers or analysts new possibilities to monitor students’ learning progress (see for example [13]; or [14]) without having to use time and resources to explicitly test students’ learning progress. Furthermore, if specific digital applications are used continuously, a more comprehensive longitudinal analysis of students’ learning progress might be possible compared to achievement tests that are maybe only taken once per year. Finally, the data generated by these digital tools, might hold information that can inform policy, educators and/or teachers on how to enhance student learning different from what we can usually learn from surveys carried out in various levels of the educational system [3].

Against this background the current paper explores what can be learned from comparing the two types of data sources discussed above in terms of potentials for measuring, monitoring and obtaining insights into reading ability and reading progress. We focus on the domain of student reading which is an essential human capital skill with important consequences for later life outcomes [15]. On the one hand we draw on a novel data set consisting of application generated data from a widely used reading app called BookBites^1^ and compare it to data from the Danish National Tests on student reading ability in grade two, four and six. BookBites (BB) offers students (grades 0-9) unlimited access to a large variety of e-books and offers reading aid (text-to-speech and dictionary) and statistics functions (words read per day, week, and month). We further take advantage of the availability of a large database of administrative registers (see [16]). The administrative registers allow for the identification of detailed parental characteristics such as parental income, profession and level of education, which are all essential factors when analyzing students’ academic performance and progress. Record linkage is achieved through the use of personal identification numbers that are available through the link with a unique user account that students use when logging into apps or test-taking systems. In the view of the foregoing, our central aim is to evaluate the potentials of the reading app data using three approaches: First, we explore the potential of the reading app generated data as an alternative measure of reading ability different from the above mentioned standardized testing systems. Second, we explore whether data from the reading app can be used to monitor reading speed over time and thus serve as a flexible real-time measure of reading speed. Third, we exploit reading app data in combination with register data on student background and reading test data to explore which factors are associated with reading speed progress. To this end, the paper will address three related research questions:

1. Can usage generated data from a reading app be used as a measure of reading ability? We explore correlations between national reading tests and user-generated reading data by comparing reading speed as measured by the reading app BookBites (BB) to reading ability test scores from a national compulsory test.
2. To what extent can usage generated data be used to monitor reading speed over time? We construct measures of reading speed progress over a nine month period (August 2019 until April 2020) and presents the results graphically.
3. What factors are related to reading speed progress and what can be learned in relation to enhancing student learning? We test several models – including machine learning models – with variables from the app generated data, student background information, national reading test scores, and classroom peer characteristics. We then interpret the relative importance of the variables.

In the following, we present analyses on these three research questions. We conclude the paper with a discussion of what kind of story the respective two different data sources tell us about student reading ability and performance. Avenues for further research and analyses will be discussed.

## Data

To test and evaluate the potential of the reading app generated data, we draw on three data sources. First, we have user generated data from a reading app, BB. We have reading app data from all student users in seven municipalities in Denmark in which the license is paid for by the municipalities. We collected data generated from August 2019 until April 2020. Through the app, students have access to almost all books written in Danish whenever they wish to read it on whatever platform they prefer (laptop, smartphone, tablet); all that is needed is an internet or data connection. The app has a list of functions like statistics on progression and supportive tools like text-to-speech and writing notes and marking text bits. Every time a student turns a page, a data entry is recorded with information on what time the page was read, how much time was spent reading the page and how many words there were on the page as well as information about the book (title, author, publisher, language, fiction/non-fiction and genre codes). Second, we have data from a mandatory national reading test for all students in 2nd, 4th and 6th grade. The reading ability tests were carried out in April 2019 (approximately five months prior to the start of the data collected from BB). The reading test score is calculated based on item-response theory using an adaptive design, meaning items are continuously adjusted to students’ proficiency level, and is comparable across grades and time (see [17], for more information). Finally, we have data from the Danish national registers (register data). In Denmark, every citizen has a unique identification number, which is given at birth. Associated with this identification number is a comprehensive centralized register with information on family members (partner, parents, kids, siblings), income (including tax reduction), educational attainment, job status, in which country you were born, your biological sex and several other pieces of information.

We use the students’ unique identification numbers for record linkage and build a new and very comprehensive data set. There are no problems in terms of selectivity or quality of the record linkage, a problem that often occurs when administrative data is linked in other contexts [18], given that all three data sources contain the unique identification number without systematic missingness. The data has been pseudonymized and is stored at Statistics Denmark, and is only accessible through a two-step login procedure and only for registered members of our research group.

In this merged data set, we have information on the students’ age, sex, ethnicity, social background (parents’ education, income and job), reading test scores and their reading habits. Together, all this information makes the data suitable for exploring the potentials and limits of user generated reading app data as a new way of measuring and monitoring students’ reading performance and progress.

We provide more information about the central variables in the next section and compare characteristics of reading app users to the general population.

### Sample description

We obtained reading app data from student users in seven municipalities in Denmark.^2^ These municipalities are among those with the highest proportion of schools with access to the reading app in Denmark. However, although the reading app, BB, is available to all students in these seven municipalities, not all students use the app. Therefore, we explore and test if the *active BB users* are different to all *potential users* from the seven municipalities and to the general *Danish population* of students in respect to central student characteristics and students’ socioeconomic background (Table 1).

**Table 1:** Comparison of means; BB-users included in analytic sample vs. BB-users in total vs. country population

Variable	Active BB users	Potential users	p-value	Danish population	p-value
Income (EURO)	47,171.73	41,527.09	0.000	39,702.70	0.000
Education (years)	14.49	14.27	0.000	13.94	0.000
Occupation (ISEI)	61.21	59.55	0.000	56.39	0.000
Test-score					
2nd grade	1.11	1.02	0.001	0.99	0.000
4th grade	0.98	0.90	0.003	0.85	0.000
6th grade	1.02	1.02	0.912	0.99	0.495
Single parents	0.20	0.21	0.027	0.21	0.004
Siblings	2.35	2.29	0.000	2.26	0.000
Immigrant status	0.15	0.13	0.000	0.12	0.000
Observations	13,999.00	54,991.00		436,436.00	

Note: Based on data from reading app, national registers and reading ability test. ISEI: International Socio-Economic Index; 0-90 [19]. Income: Household income in EURO per year.

Compared to the population of *potential users* in the seven selected municipalities, *active BB users* have parents with higher levels of education and incomes. Although the differences are statistically significant, the differences are relatively modest in terms of actual numbers (e.g. a difference of 0.22 years of education and 5644.64 EURO in difference in household income). However, the overall picture from the student background variables is that there is a degree of positive selection into usage of the BB-app, suggesting that more privileged students are more inclined to use the app. There is also a difference in reading test-scores, where the *active BB users* have a statistically significant higher score (0.08) from the remaining students, implying that either better readers use the app more or active users have better test-scores because they read more (in the app). Comparing the *active BB users* to the *Danish population* of students, the above mentioned differences appear more pronounced. However, these differences are largely driven by differences between the *potential users* from the seven municipalities and the *Danish population* of students as can be seen by comparing means from these two samples in Table 1. Yet, despite this somewhat skewed sample, the data is highly variable in respect to all included measures (see Table 2), and because part one and two of the analysis is not concerned with reporting population indices, results could be valid for all students in Denmark. As for part three of the analysis, because our sample of *active BB users* is somewhat more privileged and higher performing than the total *Danish population*, results from this part must be interpreted with some caution.

**Table 2:** Description of cleaned variables

Variable Name	mean	std	min	25%	50%	75%	max
Reading app data							
Reading speed	152.36	63.67	25.00	107.50	141.00	184.00	516.00
Session length (seconds)	64.73	43.76	7.00	31.55	53.00	87.00	274.00
Page turns per session	10.24	7.13	5	6	8	12	156
Sessions per student	19.77	36.03	1	3	8	21	559
lix	24.68	4.99	3	22	25	27	56
*Genre of book*							
Fiction	0.91	0.29	0	1	1	1	1
Non-fiction	0.01	0.09	0	0	0	0	1
Text book	0.03	0.16	0	0	0	0	1
Unlabeled	0.07	0.25	0	0	0	0	1
Weekend reading (vs. non-weekend)	0.12	0.33	0	0	0	0	1
Schools hours 7 am to 3 pm (vs. non-school hours)	0.47	0.59	0	0	0	1	1
Reading ability test data							
Reading ability test score	2.46	18.04	−58.0	−7.00	2.00	11.5	63.5
Grade level	4.85	1.41	1	4	5	6	9
Register data							
Sex (boy = 1, girl = 2)	1.54	0.50	1	1	2	2	2
Age	11.37	1.51	7	10	11	12	16
*Immigrant status*							
Both student and parents born in country	0.89	0.31	0	1	1	1	1
Born in country, but parents born abroad	0.03	0.17	0	0	0	0	1
Born abroad like the parents	0.08	0.28	0	0	0	0	1
Number of siblings	2.32	0.91	0	2	2	3	8
Single parent	0.18	0.39	0	0	0	0	1
Log income, mother	12.57	0.54	7.41	12.34	12.60	12.86	16.40
Log income, father	12.79	0.69	3.64	12.47	12.78	13.10	19.36
Years of education, mother	14.24	2.72	6.00	12.00	15.00	17.00	20.00
Years of education, father	14.00	2.82	6.00	12.00	14.00	17.00	20.00
Occupational status, mother	59.90	20.34	11.56	44.72	67.11	76.24	88.96
Occupational status, father	57.64	22.18	11.56	35.34	65.01	75.50	88.70

Note: Statistics based on reading data from August 2019 to February 2020, reading ability data and register data.

### Measures

While the administrative data and test data are readily accessible and prepared for analyses the reading app generated data needs intensive processing before it can be used as a measure of reading behavior, and subsequently, reading speed. In this section, we describe how we processed the data in order to obtain reliable measures of students’ reading speed as well as reading style. To include only valid reading application generated data, we introduced a number of criteria for inclusion. Some of these criteria were based on reading statistics such as the normal distribution of reading speed for the individual student as well as for all readers in the data, while others were formulated on the basis of an exploratory evaluation of the data at hand. We started very conservatively by introducing very broad inclusion criteria. However, evaluating the distribution of central measures, gave rise to more strict inclusion criteria and thus more data were excluded. Below we describe how we processed the data step by step and in Table 3 we show the corresponding sample sizes during this process of excluding data.

**Table 3:** Number of entries, sessions, and samples used throughout this paper. Based on reading app data

Number of	Processing step
*entries*	
22,002,522	raw data
21,995,183	removed empty/defect IDs
6,766,166	removed impossible/idle entries (1000>words_read>1 and 2000>words_per_minute>1 and 60⋅10>durations_seconds>1)
6,034,974	removed entries before August 2019 and after May 2020
5,021,219	removed words per minute less than 15 and higher than 600 (idling and skipping)
4,689,763	removed reading speed outliers according to 1 ⋅ interquartile range
4,462,661	removed words per minute outliers according to 1.5 ⋅ interquartile range
4,218,061	removed words read outliers according to 1.5 ⋅ interquartile range
4,039,967	removed seconds duration outliers according to 1 ⋅ interquartile range
2,157,156	after session detection, see Sect. 2.2
*sessions*	
215,521	total sessions detected, see Sect. 2.2
22,056	for comparison with national test used in Sect. 3.1 (only users: with more than 10 sessions; in grade 3, 5, and 7; active in September to November 2019)
209,952	for improvement over time used in Sect. 3.2 (removed users that have fewer than four entries in four months)
*samples*	session aggregates per user
1542	for model to predict reading speed in Sect. 3.3
1236	training data used to fit the models
308	test data to evaluate the models

The BB reading app generates data every time a student turns a page. That means that reading activity is recorded as page turns while a student is reading a book, one page at a time. Our full data on page turns consists of 22,002,522 entries. However, data is also recorded when a student is flipping back and forth in a book to obtain an overview of the content or when a student opens a page in a book and then spends 20 minutes on some social media simultaneously, when the student flips pages to locate some specific text passage and when a student opens a number of different books to get a sense of whether or not the book seems interesting. Furthermore, because the BB license is paid for by the municipality, and there are nearly unlimited books available, the students’ parents also occasionally use the app for book reading (this information was given to us by the BB company owner and is confirmed by the reading speed and book titles of selected reading sessions). As a first step to handle this irregular page turn data, we processed the data into reading sessions. We register a reading session if 5 or more pages are turned in the same book. Page turns are only included if reading time per page is above 10 seconds and below 10 minutes and is otherwise considered as unreliable page turns. Further, the reading speed between any two consecutive page turns is only allowed to differ by 75 words per minute. One student can thus have several reading sessions on one day. We have access to reading sessions in the time period from 11.08.2019 to 20.05.2020.

Reading sessions are throughout the analysis used as the basis on which to derive central measures of interest. We have 215,521 reading sessions generated by 10,903 students from 194 schools. On average the students have 19.77 reading sessions (median: 8; min: 1; max; 559) associated.

#### Reading speed, reading speed progress and reading ability

The central variables of interest are reading speed, reading speed progress, and reading ability. Reading speed is calculated on the basis of the number of words per page divided by how much time a student spends reading that page. The information about words per page and time spent is accessible from the usage generated reading app data. To increase the reliability and the robustness of the measure, we use the median reading speed of each reading session. Every student thus has one measure of reading speed per session.

Reading speed progress is calculated using the reading speed in combination with the time series structure of the reading app data and is thus a measure of the difference in reading speed between two consecutive months. By graphing reading speed over time, we evaluate how pronounced reading speed progress is over this relative short period of time. Reading speed progress can thus be either negative, zero or positive.

Reading ability is calculated on the basis on a nation wide compulsory reading ability test [17]. Here the three sub domains language comprehension, decoding and reading comprehension are tested. The test is based on item response theory and the score has been standardized with a mean and standard deviation of one. We follow Beuchert and Nandrup [17] and construct a measure of reading ability by calculating the mean score of these three sub domains.

Besides these three central measures, we use numerous other information in our analysis. The selection of most of the variables was based on knowledge from the existing literature within the field of educational research in general and research on reading specifically. However, we also included a few variables that to the best of our knowledge have not been used in such analyses before. These are exclusively the variables derived from the app generated data. In Table 2, we present the included variables.

Derived from the *reading app data*, besides the variable *reading speed*, we include *session length* in seconds, *page turns per session*, *sessions per student*, *lix* (which is a measure of text difficulty – higher numbers indicating more difficult text [20]), genre of book (*fiction, non-fiction, text book or unlabeled*),^3^ whether the reading is done on weekends (*weekend reading* vs. non-weekend reading) and whether the reading session is finished in or out of normal school hours (*school hours* (7 am to 3 pm) vs. non-school hours). We have not found literature including these variables in models examining reading ability or reading speed progress. Most of the existing literature focus on either brain activity and neural circuits [21], on various non-cognitive skills [22] or on closely related sub-dimensions of reading such as linguistic comprehension and narrative skills [23] to predict reading ability or reading speed progress.

From the *reading ability test data*, besides the *reading ability test score* we use information on *grade level*. In part one and two of the analyses, we test the potential of the reading app generated data by grade and in the third part of the analyses, we anticipate that reading speed progress might vary by grade level.

From the *register data* we obtain information on student *sex* (boy = 1, girl = 2), *age*, their immigrant status (*both student and parents born in country, born in country, but parents born abroad, and born abroad like the parents*), *number of siblings* and whether the students lives with one or two parents (*single parent* vs. two parents).

Furthermore, we have data on parental background including mother’s and father’s income^4^ (we use the logarithm: *log income, mother and father* respectively), *years of education, mother and father* respectively and *occupational status, mother and father* respectively, using the International Socio-Economic Index (ISEI) scale [19]. We know from existing literature on reading ability and on scholastic abilities in general, that student characteristics and family background are strong predictors of performance and educational success (see e.g. [24]). We include sex and parental educational background in part one of the analyses and all the above variables in part three of the analyses. In Table 5(c), we tranform years of education into values from 1-7 based on the International Standard Classification of Education (ISCED).^5^

In Table 2, we present descriptive statistics for the selected variables. All information in Table 2 are based on individual and family background characteristics. However, being a student in a specific school, in a specific classroom and having specific classmates is not an isolated experience. Students are affected by their peers and vice versa in the classroom [25]. To take these peer influences into account in our analysis in the third part of this paper, we include a number of measures derived from the above mentioned variables calculated on the basis of peers. The difference is calculated by subtracting the median of the group (classroom or grade) and dividing by the median absolute deviation, which is a robust way to standardize the data by its group values. First we use lix, reading speed, reading ability score, length of reading sessions, number of reading sessions, and reading speed progress relative to peers in class. Additionally, we include a few measures indicating whether the student is an ambitious reader in absolute terms by comparing his/her level to grade level averages. These are lix, number of reading sessions and length of reading sessions relative to grade level. These measures are listed in Table 4.

**Table 4:** List of peer variables

Variable Name
Reading speed relative to class
Session length (seconds) relative to class
Sessions per student relative to class
Lix relative to class
Reading ability test score relative to class
Reading speed progress relative to class
Lix relative to grade
Sessions per student relative to grade
Session length (seconds) relative to grade

Note: All variables in this table are derived from the variables in Table 2. Class refers to the students in the specific classroom the student attends whereas grade refers to all BB users in the same grade level as the student.

### Methods and analytical plan

In the first part of the analyses, we compared *reading ability test scores* from the formal testing system to the *reading speed* measures derived from the BB app. In this comparison, we used reading app data from grade three, five and seven. For these students, the most recent results are based on the national reading ability test that was carried out in April 2019, which means that the test were taken while the students were still in grade two, four and six. Because the reading ability tests were carried out in April, we compare the reading ability test scores to reading app data generated closest to that time, namely reading app data from September through November 2019 to have the most concurrent and thus comparable data. We use the average reading speed of individual students from these three months and exclude students with less than ten reading sessions within this time period. Subsequently, we present the results by reporting the correlation coefficients and depict the results using kernel density estimate plots.

Because this article is preoccupied with the potential of reading app generated data and therefore also concerned with how to approach and handle such data, we included an example of how our first analysis looked before introducing the more strict inclusion criteria presented in Sect. 2.2.

To explore the potential for evaluating reading speed progress using reading app data, we draw a graph showing the average reading speed per month per grade and discuss the results.

In the third and final part of the analyses, we test several models and their performance in identifying key factors associated with reading speed progress. We include all variables from Table 2 and 4 in the models and evaluate their performance in terms of their level of precision. Then we evaluate the significance of the variables one by one, to present a list of variables that seemingly relates to reading speed progress.

## Results

### National test versus reading speed

**Figure 1:**
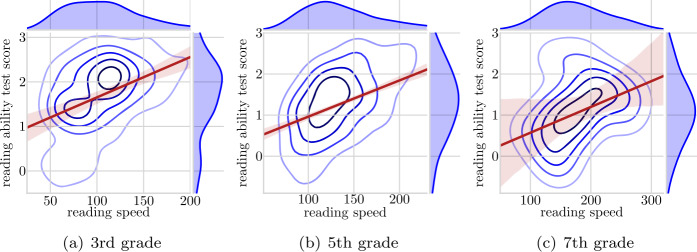
Kernel density plots, predicted line and confidence intervals for the relationship between average reading speed and reading ability test scores on the cleaned data. Grades three, five and seven. Reading speed from September through November 2019. Reading ability test scores from April 2019.

In Fig. 1, we show the results of the correlation between reading ability test scores and reading speed data from the reading app. We present one figure from each of the grades three, five and seven respectively and we use kernel density estimate plots to obtain a more fine-grained view of the association between the two variables.

The predicted regression lines for all three grade levels indicate a positive relationship between the student’s reading ability test score and our calculated measure of reading speed. Furthermore, the distribution and density of plots indicate that reading speed is higher in higher grades. Please note that the reading ability score does not seem to improve. This is due to the fact that the reading ability test is age adjusted.

**Figure 2:**
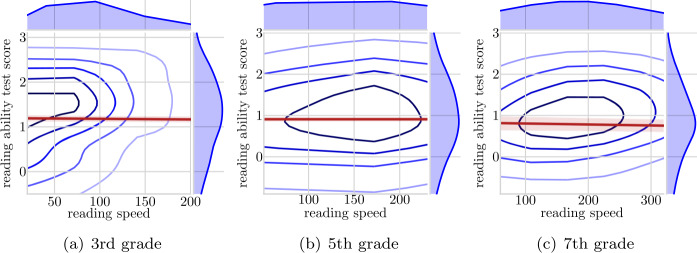
Kernel density plots, predicted line and confidence intervals for the relationship between average reading speed and reading ability test scores on all the data (prior to inclusion criteria). Grades three, five and seven. Reading speed from September through November 2019. Reading ability test scores from April 2019

To illustrate the state of the reading app data before implementing our inclusion criteria presented in Sect. 2.2, we illustrate in Fig. 2 how the correlation between the reading ability score and reading speed would have looked like if these were not introduced. Clearly, app generated data are not readily accessible for drawing indices about real world reading.

In Table 5, we show that the correlation coefficients for the comparison of reading ability test scores and reading speed. These range from 0.39 in grade three to 0.48 in grade five. Assuming the reading ability test score is valid and reliable, correlation coefficients of the given size indicate that the reading app usage generated reading speed measure only to some extent is a reliable measure of reading ability as measured by the national reading ability test. However, at least three conditions must be taken into account in the evaluation of the reliability of our reading speed measure for indices about reading ability. First, as described earlier, the national reading ability test measures three components, language comprehension, decoding and reading comprehension, none of which is equal to reading speed. As such, our measure of reading speed is to some extend measuring a different skill than the reading ability test does [26]. Second, the reading ability test was conducted in April 2019, while the reading app usage data which we use for constructing a reading speed measure was generated from September to November 2019 – that is five to seven month later. As such, the test of the reliability of our reading speed measure is far from perfect. Data on actual reading speed that were measured synchronous to the reading in the app, would therefore most likely improve the evaluation of app usage generated reading speed data. Finally, given that the reading ability test is only carried out every second year in a non-natural testing setup one might argue that reading speed data from the app is the most reliable measure of the actual everyday reading ability of the students.

**Table 5:** Correlation between reading ability test data and app generated reading speed

(a) By grade.			
Grade	Correlation	# students	# sessions
3rd	0.386	172	4510
5th	0.478	562	17,043
7th	0.448	17	503

(b) By grade with the outlier data.			
Grade	Correlation	# students	# sessions
3rd	0.049	915	158,749
5th	0.031	1831	198,256
7th	−0.020	150	8998

(c) By grade and parental educational level (edu). Parental educational level is coded from 1 (low) to 7 (highest) in accordance with isced 1997.				
Grade	Edu	Correlation	# students	# sessions
3rd	3	0.080	28	817
	4	0.505	6	170
	5	0.419	34	715
	6	0.475	81	2291
	7	0.008	23	517
5th	1	NaN	<5	NaN
	2	0.023	22	526
	3	0.294	141	3921
	4	0.491	31	1131
	5	0.380	174	5546
	6	0.590	155	4856
	7	0.746	33	981
7th	3	0.349	6	201
	4	NaN	<5	NaN
	5	NaN	<5	NaN
	6	0.386	7	227
	7	NaN	<5	NaN

(d) By grade and parental income. Parental income is split into below and above the median income in Denmark.				
Grade	Low income	Correlation	# students	# sessions
3rd	High income	0.393	132	3390
	Low income	0.360	40	1120
5th	High income	0.522	359	11,179
	Low income	0.402	203	5864
7th	High income	0.295	10	337
	Low income	0.655	7	166

(e) By grade and sex.				
Grade	Sex	Correlation	# students	# sessions
3rd	Boys	0.330	80	2288
	Girls	0.456	92	2222
5th	Boys	0.596	247	6902
	Girls	0.380	315	10,141
7th	Boys	0.604	10	360
	Girls	0.019	7	143

We also present the correlation coefficients of the reading app generated data before introducing inclusion criteria. These are shown in Table 5(b) and clearly, using the full data conceals the correlation between reading ability test scores and reading speed.

In Table 5(c), 5(d) and 5(e), we split the sample in accordance with parental educational background, household income and student sex respectively. To secure anonymisation of individual students, we only included the correlation coefficients if there were more than five BB users in a specific group. As shown in the tables, the amount of data is somewhat limited; especially for grade 7 but also to some extend for grade 3. As a consequence, we focus on grade 5 in our interpretation and when discussing the results. The results indicate that the correlation between reading ability test scores and the measure of reading speed for students with higher educated parents has a higher correlation (Table 5(c)). This is true for 5th grade with a positive and almost linear relationship between the educational level of parents and the size of the correlation between reading ability test score and reading speed. Based on a very limited amount of data, results regarding grade 3 indicate that the same relationship might exist for this group of students too. There is not enough data in grade 7 to interpret the results. One plausible explanation for these results would be if students with higher educated parents are more stable in terms of performing in the test and their reading speed. Meaning the underlying trait, reading ability, measured by both the formal test and the user-generated data, varies less over time for students with higher educated parents. We know from test results that students with higher educated parents are better readers, on average [27], which might also help explain the result. Specifically, the reading speed may be measured more precisely for better readers. Table 5(d) shows that students from high income homes are associated with the highest correlation coefficient. Again, this result is most pronounced for grade 5, where we also have the most data, and less pronounced for grade 3. Based on very few reading sessions and students the results are opposite for grade 7. Overall, it seems that the correlation between reading ability test scores and reading speed is larger in magnitude for students from high income homes than for student from low income homes. Like for parental education, higher income is, on average, associated with higher performance and the explanation presented just before might very well once again be relevant. Finally, when it comes to student sex, the results are mixed. In grade 3, girls are the ones with the highest correlation coefficient whereas in grade 5, boys are the ones with the highest correlation coefficient (Table 5(e)). The results concerning grade 5 are surprising, because girls are on average better readers than boys,^6^ and this result therefore indicates that the higher correlation coefficient is not necessarily a result of students being better readers.

Overall, the coefficients of the correlation between reading ability test scores and reading speed indicate that the app generated data has potential for measuring reading ability in terms of reading speed. However, when compared to reading test scores, the measure of reading speed is far from perfect.

### Reading app generated data to measure student reading speed over time

**Figure 3:**
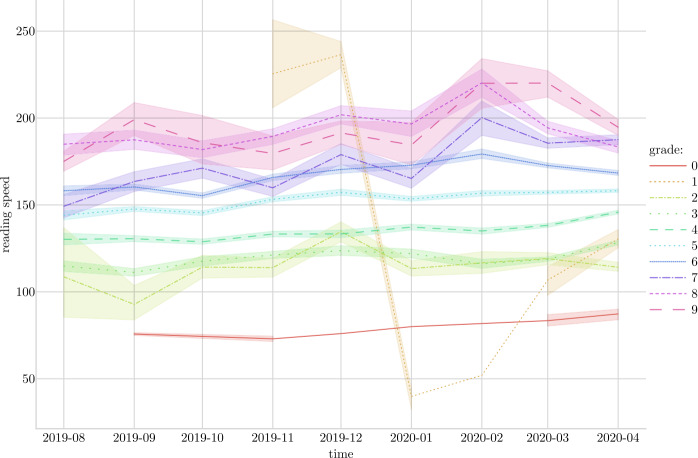
Reading speed over time per grade level. Regression line based on reading speed averages. With confidence intervals

In this part of the analyses, we evaluate the potential of the reading app generated data by testing to which extend it can measure reading speed over time and show reading speed progress. Results for all grades are shown in Fig. 3 and there is an indication of a slight upwards trend for most of the grades in terms of reading speed. The fact that we have very little data from grades zero, one and two and for grade seven, eight and nine helps explain the zigzagged progression lines as well as the relatively large confidence intervals for those grade-levels. Looking at only grades three to six, where we have more reading sessions and thus more data, the reading speed progression is more pronounced. This steady progress suggests that our measure of reading speed progress across consecutive months can capture small but meaningful increases in reading speed.

**Figure 4:**
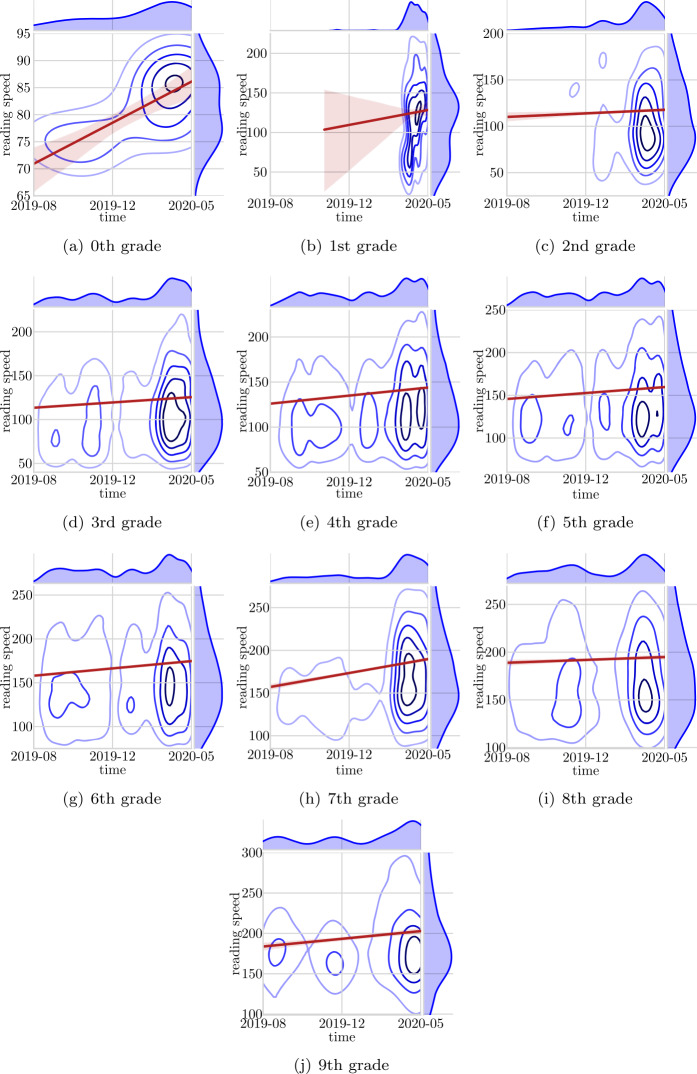
Kernel density plots, predicted line and confidence intervals for reading speed over time. Per grade

Figure 4 shows kernel density plots for all grades and the same upwards trend for most grades is apparent. The lack of reading data is also highlighted, and the associated distribution curve brings to light the difference in reading app activity before and during closure of schools due to the Covid-19 pandemic. Overall, these results indicate that using reading app generated data has the potential to monitor reading speed progress. However, at the same time these results highlight the need for a sufficient amount of data before reading speed progression can be measured accurately.

### Reading speed progress

We use reading app data from August 2019 to January 2020 in pairs of three months, two of which is input month, and the third is target. Note that we used the variables from the second month as is and additionally generated a *progress* variable for all non-static variables by subtracting the first from the second month (e.g., the *reading speed progress variable* depicts the change over the two input months). For example, we use reading speed progress from August to September to predict reading speed progress from September to October. We use this subset of the data to train our models and to identify associations with reading speed progress in February 2020.^7^ In addition, we employ the static data from the national reading ability test and register data. In other words, the data is in a wide format, with one observation per student and non-static variables appear for each month as well as for change between months (in pairs of three months). As a result and in addition to the variables that are listed in Sect. 2.2 on measures, we include the following variables based on the preceding months: *last reading speed, last reading speed relative to class, reading speed progress, last reading speed progress, and last reading speed progress relative to class*. To center and scale the data we used the median and interquartile range (0.25 and 0.75) based on the training data, respectively.

We decided to use two models: linear regression and ridge regression [29]. In particular, ridge regression was chosen since it applies l2 regularization to deal with high correlations and is able to handle variables with multicollinearity. We also tried non-linear methods such as support vector regression (SVR) [30] with a linear as well as a Gaussian kernel, random forest [31], extremely randomized trees [32], and LightGBM [33], but the model results were not better than the linear models. All model parameters were tuned via a grid search over a wide selection of parameters, specific to each method. To avoid overfitting, we conducted the parameter search solely on the training set through 5-fold cross validation. We accounted for the temporal aspect of the data by using temporal splits (sliding window approach) to avoid using future data in fitting the models. We did not consider random intercept models on a class level, since we would need to carefully split our data to keep a clean separation between training and test data, which would reduce our data even more (not all classes had multiple students). The results can be seen in Table 6. Since linear models performed better than the non-linear models, we choose them for our analyses. An additional benefit is that linear models are easier to interpret.

**Table 6:** Performance comparison of all models on the training and testing set. The $R^{2}$ score and root mean squared error (RMSE) indicate how well we can fit the true reading progress. Accuracy measures if the direction of progress is correctly predicted and the f1 score is a combination of precision and recall. Since both, accuracy and f1 score are the same, we know that precision (positive predictive value) and recall (sensitivity) have the same value. For all, except RSME, holds that higher values indicate a better fit. Test and train values are close to one another, which suggests that no strong over-fitting occurred. We finally chose linear regression and ridge regression as final models

Split	Metric name	Accuracy	$R^{2}$	RMSE	f1
Test	linear regression	0.621	0.159	16.400	0.621
	ridge regression	0.621	0.180	16.197	0.621
	extremely randomized trees	0.568	0.114	16.743	0.568
	LightGBM	0.636	0.141	16.492	0.636
	random forest	0.575	0.086	17.005	0.575
	SVR (RBF kernel)	0.594	0.136	16.534	0.594
	SVR (linear kernel)	0.620	0.157	16.338	0.620
Train	linear regression	0.648	0.158	16.568	0.648
	ridge regression	0.642	0.167	16.481	0.642
	extremely randomized trees	0.680	0.293	14.968	0.680
	LightGBM	0.660	0.197	15.951	0.660
	random forest	0.698	0.403	13.751	0.698
	SVR (Gaussian kernel)	0.657	0.193	15.990	0.657
	SVR (linear kernel)	0.663	0.172	16.198	0.663

**Figure 5:**
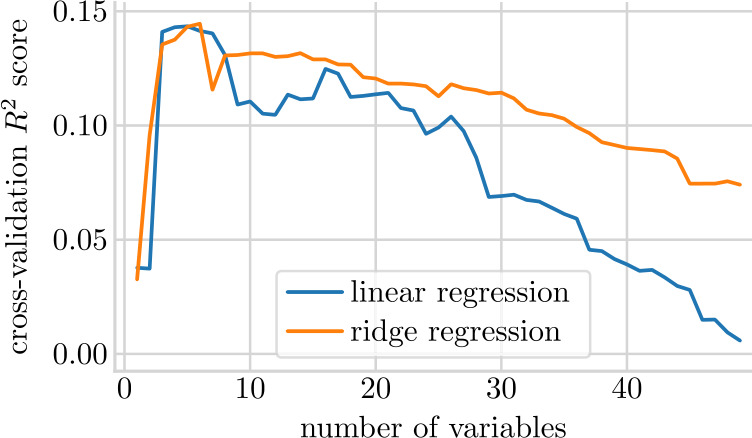
Performance of models (linear regression and ridge regression) on the training set while slowly removing variables

To reduce the number of variables, we applied recursive feature elimination (RFE) [34] to linear regression and ridge regression. The results are shown in Fig. 5. This technique removes one feature at a time based on the p-value (testing if a variable is relevant), fits the model again, and saves the $R^{2}$ score. We choose the p-value instead of relying on coefficients alone since we have both, continuous and categorical variables. Finally, the configuration with the highest $R^{2}$ is chosen for the final model. Both models show the highest performance with only a few variables selected (3-8).

**Figure 6:**
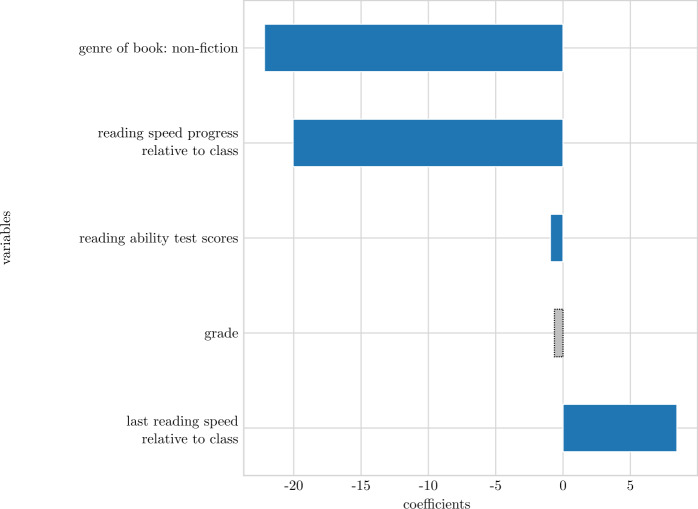
Standardized coefficients of linear regression model with reading speed progress as outcome. Non-significant influences ($p < 0.05$) are marked by grey bars with dotted borders

**Figure 7:**
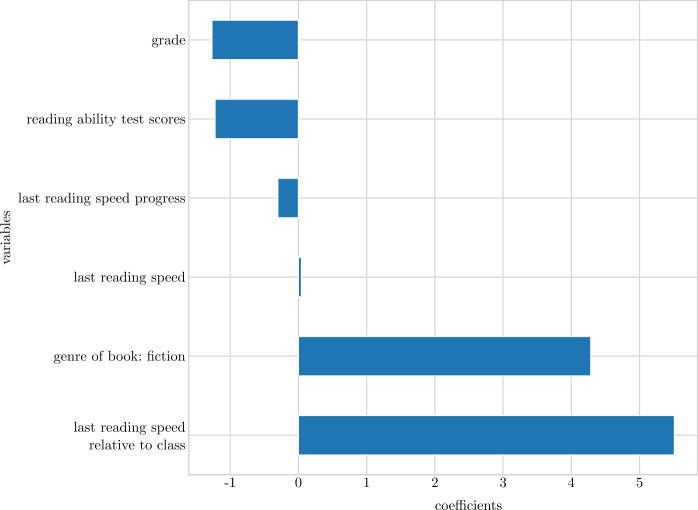
Standardized coefficients of ridge regression model with reading speed progress as outcome. Non-significant influences ($p < 0.05$) are marked by grey bars with dotted borders

In Table 7, all coefficients, *t*- and p- values of the variables that were left in the model after reducing the number of variables are shown. The coefficients are standardized since the data was standardized via robust measure before creating the models (interquartile range scaling for continuous values and between −1 and 1 for categorical values). The coefficients of this table are also shown in Fig. 6 and 7 and sorted by coefficient values. Note that the magnitude of coefficients is not comparable across models, more interesting is the selection and sign of variables. Coefficients that have associated p-values below 0.05 are in blue colour, whereas the rest are grey colored with dotted borders. The results point to several variables associated with reading speed progress. In the linear regression model (Fig. 6), the proportion of reading in *non-fiction* books is negatively associated with reading speed progress. Also the *reading speed progress relative to class* has a large negative coefficient indicating that students that until now have had a large reading speed progress relative to peers in the class are negatively associated with future reading speed progress. Furthermore, the higher a student scored on the national *reading ability test*, the smaller a reading speed progress is predicted by this model. i.e.slow readers are associated with the largest reading speed progress. This might be the result of a ceiling effect. When a student reaches a certain (unknown) reading speed, the student will not be able to show much progress in terms of reading speed. Finally, students that reads fast relative to the peers in the class are associated with the largest reading speed progress gains.

**Table 7:** Standardized coefficients from the linear and ridge regression models on *reading speed progress* and associated *t*- and p-values.

Model	Variable	Coefficient	*t*	p
Linear regression	reading ability test score	−0.942	−2.362	0.018
	grade	−0.638	−1.384	0.167
	genre of book: non-fiction	−22.180	−3.519	0.000
	last reading speed relative to class	8.460	9.759	0.000
	reading speed progress relative to class	−20.050	−12.670	0.000
Ridge regression	reading ability test score	−1.222	−3.034	0.002
	genre of book: fiction	4.285	2.252	0.025
	grade	−1.267	−2.435	0.015
	last reading speed	0.044	3.208	0.001
	last reading speed relative to class	5.510	4.089	0.000
	reading speed progress	−0.302	−12.794	0.000

Note: Statistics based on reading data from August 2019 to February 2020, reading ability data and register data.

The ridge regression model also highlights *reading ability test scores*, *reading speed relative to peers*, and genre of the books being read (here the proportion of *fiction* read is positively associated with reading speed progress) as being significantly associated with reading speed progress. Additionally, *grade* level is highlighted as being related to reading speed progress. The higher a grade level students attend, the slower the progress. Again, this might be a consequence of the before-mentioned ceiling effect when it comes to reading speed. Students in higher grades are closer to their unknown maximum reading speed potential, and there is not much room for improvements. Also previous *reading speed progress* is negatively associated with subsequent reading speed progress. If a student showed great improvement in terms of reading speed progress, the model predicts a decline in progress going forward. As such, a steep reading speed progress curve is most likely to flatten out in the time that follows. Finally, *reading speed* in the preceding month has a small but statistical significant positive association with reading speed progress.

Overall, it seem that the *last reading speed relative to class* is very important in both models. As such, it is more important whether a student reads fast relative to the peers in the classroom, rather than if the student reads fast in absolute terms when it comes to reading speed progress. The genre of the books being read is also highlighted as being significant in both models. One explanation could be that the universe that is offered to readers of fictive books to a larger extend invites readers into a state of flow when reading – as opposed to readers of non-fiction – and this state of flow enhances reading speed progress. Another possible explanation is that struggling readers tend to pick non-fiction books, whereas other students more often pick fiction books. Finally, test scores from the national reading ability test is related to reading speed progress in both models. This indicates that students with a lower reading abilities are more likely to increase their reading speed than high ability readers.

## Conclusion and discussion

In this paper we tested and evaluated the potential of usage generated data from digital learning apps used by school children. First we compared reading speed as measured by the reading app BookBites (BB) to reading ability test scores from a national compulsory test. The results showed that the reading app data holds, to some degree, the potential to measure one aspect of reading ability namely reading speed. However, the correlation coefficients were only moderate. A clear limitation of this analysis is that we have asynchronous measures in terms of timing and that the reading ability test is not measuring reading speed per se but other components of reading ability. Furthermore, the analysis showed that correlation coefficients are higher for boys than for girls and higher for students with higher levels of education for their parents and for students with parents with an above median income.

So far, we have anticipated that the nationally administrated reading ability test is a valid and reliable measure of students’ reading ability. If we, contrary to this fundamental assumption, assume that the national reading ability test is not a precise measure,^8^ and that the reading speed measure is highly reliable, results from Table 5(c), 5(d) and 5(e) can be interpreted in an utterly different way. The correlation coefficients then indicate that the national reading ability test is a more reliable measure for male students with highly educated parents with an above median income. If this interpretation is correct, our results point to relevant problems in regards to the reading test both from a standpoint of monitoring student progress as well as from a perspective of social inequality – given that test results for girls and students with less educated parents would potentially be misleading.

However, as discussed in the *National test versus reading speed* section, more research and better data is needed in order to draw such a conclusion.

Second, we tested if the reading app generated data could be used to monitor reading speed over time, and thus evaluate reading speed progress. While the analysis highlighted that such analysis requires lots of data, it also revealed that there was such a potential. Especially for grades three to six, where there was most reading activity in the data, the analysis was able to detect a reading speed progress over the period of time where we have data.

Third and finally, we tested several models by training them to identify associations between selected variables and reading speed progress. Two models, Ridge and linear regression, were chosen based on their level of explained variance ($R^{2}$’s of 0.180 and 0.159 respectively). Several variables were included in the two models because of their significance in terms of explaining variance in reading speed progress. Among these were genre of the book, grade level, reading ability test score and some measures relative to peers in class. While the results are not to be interpreted as causal, the analysis revealed an unseen potential of reading app generated data to inform policy and teachers in new ways to enhance students learning. For example, the large coefficient on students’ reading speed relative to the reading speed of peers, directs our attention to the theory of being a ‘big fish in a little pond’ [35] and contextual attributes of the classroom environment. The coefficient support this theory by indicating that being a capable reader relative to ones peers is associated with reading speed progress. Also reading more often in fiction as opposed to reading non-fiction is associated with reading speed progress.

Based on these three analyses, we conclude by stating that it seems that formal data on reading ability and application generated reading data in many ways tell two sides of the same story. However, while most formal reading ability test are only conducted once every year, the time series nature of the reading app data has proven in the analyses to hold great potential. However, the analyses did also show that large amounts of data are needed to produce reliable results, and as a consequence, the potential of producing reliable measures on the individual students level is at best problematic.

Future avenues for analyzing the potential of usage generated learning app data are many. One is related to finding the right text-difficulty for students to read. To this end, text-difficulty is usually calculated using a text-based approach (objective). Using reading app generated data opens the possibility to mark books with performance-based (behavioural) recommendations or even a combination of both (subjective) to offer the individual student guidance in whether a text is easy, intermediate or hard to read for that particular student [36]. Also looking at application generated data from different subjects e.g. mathematics could be interesting as well as looking at other levels in the education system. Finally, the data could be even more enhanced by learning more about the context of the classroom environment. If, for example, one could identify teacher assigned books, analyses could become even more precise.

## Data Availability

The datasets analysed during the current study are not publicly available as they hold sensitive personal information.

## Footnotes

1 https://bookbites.com/en/

2 We made data agreements with eight municipalities, but one cancelled their contract with BB.

3 We use the four genre categories as separate variables based on the share of total reading sessions spent on the specific genre.

4 Total net income from all sources including the rental value of their home. After taxes and deduction.

5 https://ec.europa.eu/eurostat/statistics-explained/index.php/International_Standard_Classification_of_Education_(ISCED)

6 http://timssandpirls.bc.edu/pirls2016/international-results/pirls/student-achievement/reading-achievement-by-gender/

7 Because reading data from March 2020 seems to vary in systematic ways from the reading data before schools closed down due to the Covid-19 outbreak [28] we chose not to use data from March in this analysis

8 The reading ability test has been shown to have a number of problems in relation to both the validity and the reliability [7]

## References

[1] Sellar S, Thompson G, Rutkowski D (2017). The global education race: taking the measure of PISA and international testing.

[2] Hursh D (2005). The growth of high-stakes testing in the usa: accountability, markets and the decline in educational equality. Br Educ Res J.

[3] Williamson B (2017). Big data in education: the digital future of learning, policy and practice.

[4] Ministry of Children and Education (2020) Evaluation, Tests & Student Plans. https://eng.uvm.dk/primary-and-lower-secondary-education/the-folkeskole/evaluation-tests-student-and-plans. Accessed 2020-10-09

[5] Commission E (2018). The 2018 international computer and information literacy study (ICILS).

[6] Rambøll, Boston Consulting Group (2014) Anvendelse af digitale læremidler [the use of digital teaching tools]. Technical Report September, Rambøll and Boston Consulting Group, Copenhagen

[7] Flarup LH (2020) Evalueringen Af de Nationale Test [Evaluating the National Tests] Copenhagen, Denmark pp 1–31. VIVE - the Danish center for social science research. https://www.vive.dk/da/udgivelser/evalueringen-af-de-nationale-test-14769/

[8] von der Embse N, Hasson R (2012). Test anxiety and high-stakes test performance between school settings: implications for educators. Prev Sch Fail Altern Educ Child Youth.

[9] Harlen W (2004). A systematic review of research evidence of the reliability and validity of assessment by teachers used for summative purposes.

[10] Sung Y-T, Chang K-E, Liu T-C (2016). The effects of integrating mobile devices with teaching and learning on students’ learning performance: a meta-analysis and research synthesis. Comput Educ.

[11] Moody AK, Justice LM, Cabell SQ (2010). Electronic versus traditional storybooks: relative influence on preschool children’s engagement and communication. J Early Childh Literacy.

[12] Domingues-Montanari S (2017). Clinical and psychological effects of excessive screen time on children. J Paediatr Child Health.

[13] Long P, Siemens G (2011) Penetrating the fog: analytics in learning and education. Educause, 31–40

[14] Hwang G-J, Chu H-C, Yin C (2017). Objectives, methodologies and research issues of learning analytics. Interact Learn Environ.

[15] Ritchie SJ, Bates TC (2013). Enduring links from childhood mathematics and reading achievement to adult socioeconomic status. Psychol Sci.

[16] Jensen VM, Rasmussen AW (2011). Danish education registers. Scand J Public Health.

[17] Beuchert L, Nandrup A (2018) The danish national tests at a glance. Nationaløkon Tidsskr [Danish Journal of Economics] 2

[18] Sakshaug JW, Antoni M, Sauckel R (2017). The quality and selectivity of linking federal administrative records to respondents and nonrespondents in a general population survey in Germany. Surv Res Methods.

[19] Ganzeboom HBG, Treiman DJ (2010). International stratification and mobility file: conversion tools.

[20] Björnsson CH (1968). Läsbarhet [readability].

[21] McNorgan C, Alvarez A, Bhullar A, Gayda J, Booth JR (2011). Prediction of reading skill several years later depends on age and brain region: implications for developmental models of reading. J Neurosci.

[22] Nicolau CC, Navas ALGP (2015). Assessment of skills that predict reading success in 1st- and 2nd-grade children of elementary school. Rev CEFAC.

[23] Babayiğit S, Roulstone S, Wren Y (2021). Linguistic comprehension and narrative skills predict reading ability: a 9-year longitudinal study. Br J Educ Psychol.

[24] Gamoran A, Long DAL, Teese R, Lamb S, Duru-Bellat M (2007). Equality of educational opportunity a 40 year retrospective. International studies in educational inequality, theory and policy.

[25] Sacerdote B, Hanushek EA, Machin S, Woessmann L (2011). Chap. 4 – peer effects in education: how might they work, how big are they and how much do we know thus far?. Handbook of the economics of education.

[26] Jackson MD, McClelland JL (1979). Processing determinants of reading speed. J Exp Psychol Gen.

[27] OECD (2018). Breaking down barriers to social mobility (PISA).

[28] Reimer D, Smith E, Andersen I G, Sortkær B (2021). What happens when schools shut down? Investigating inequality in students’ reading behavior during Covid-19 in Denmark. Res Soc Stratif Mobil.

[29] Hoerl AE, Kennard RW (1970). Ridge regression: biased estimation for nonorthogonal problems. Technometrics.

[30] Drucker H, Burges CJ, Kaufman L, Smola A, Vapnik V (1997). Support vector regression machines. Adv Neural Inf Process Syst.

[31] Breiman L (2001). Random forests. Mach Learn.

[32] Geurts P, Ernst D, Wehenkel L (2006). Extremely randomized trees. Mach Learn.

[33] Ke G, Meng Q, Finley T, Wang T, Chen W, Ma W, Ye Q, Liu T-Y (2017). Lightgbm: a highly efficient gradient boosting decision tree. Adv Neural Inf Process Syst.

[34] Guyon I, Weston J, Barnhill S, Vapnik V (2002). Gene selection for cancer classification using support vector machines. Mach Learn.

[35] Marsh HW, Parker JW (1984). Determinants of student self-concept: is it better to be a relatively large fish in a small pond even if you don’t learn to swim as well?. J Pers Soc Psychol.

[36] Tamor L (1981). Subjective text difficulty: an alternative approach to defining the difficulty level of written text. J Read Behav.

